# Periodontal Proteomics: Wonders Never Cease!

**DOI:** 10.1155/2013/850235

**Published:** 2013-12-31

**Authors:** Harpreet Singh Grover, Shalini Kapoor, Neha Saksena

**Affiliations:** Department of Periodontology, Faculty of Dental Sciences, SGT University, Budhera, Gurgaon, Haryana 122505, India

## Abstract

Proteins are vital parts of living organisms, as they are integral components of the physiological metabolic pathways of cells. Periodontal tissues comprise multicompartmental groups of interacting cells and matrices that provide continuous support, attachment, proprioception, and physical protection for the teeth. The proteome map, that is, complete catalogue of the matrix and cellular proteins expressed in alveolar bone, cementum, periodontal ligament, and gingiva, is to be explored for more in-depth understanding of periodontium. The ongoing research to understand the signalling pathways that allow cells to divide, differentiate, and die in controlled manner has brought us to the era of proteomics. Proteomics is defined as the study of all proteins including their relative abundance, distribution, posttranslational modifications, functions, and interactions with other macromolecules, in a given cell or organism within a given environment and at a specific stage in the cell cycle. Its application to periodontal science can be used to monitor health status, disease onset, treatment response, and outcome. Proteomics can offer answers to critical, unresolved questions such as the biological basis for the heterogeneity in gingival, alveolar bone, and cemental cell populations.

## 1. Introduction

Periodontal tissues comprise multicompartmental groups of interacting cells and matrices that provide continuous support, attachment, proprioception, and physical protection for the teeth [1]. The complex interactions of cells and matrix within compartmental groups make the molecular understanding of periodontium [1]. Visual examination, tactile examination, periodontal pocket depth, clinical attachment level, and various periodontal indices are basis of periodontal diagnosis in day to day clinical practice [2]. To develop screening and diagnostic modalities for early detection of periodontal disease is the need of hour. To achieve that, it is important to understand the underlying science and molecular basis of tissue complexity of periodontium. Evolvement with time has brought biomarkers, proteomics, genomics, and metabolomics in the forefront for periodontal diagnosis as well to assess response to therapy [1, 3, 4]. For more in-depth understanding of periodontium, its proteome map, that is, complete catalogue of the matrix and cellular proteins, is required.

Proteins are essential part of the metabolic pathways of a living cell and its entire set with the modifications, produced by an organism or system, is considered a proteome. The term “proteomics” is a blend of “protein” and “genome” [5] was first coined in the year 1997 by James to make an analogy with genomics, the study of the genes [6]. In simple terms, proteomics is defined as the study of all proteins present in a particular cell or an organism in a given environment and at a specific stage in the cell cycle [7]. Proteome analysis of bone and dental structure (enamel, periodontal ligament, and cementum) and oral fluid diagnostics (saliva and GCF) are the primary areas where dental proteomics has shown promising outcomes [7].

A paradigm shift for periodontal science occurred which is used to

- monitor onset of disease,
- monitor status of disease in regard to health,
- monitor response to treatment,
- monitor outcome.

The major challenge for research workers in periodontology is to embrace proteomics approaches when appropriate and start to apply them to critical, unresolved questions such as molecular and biologic understanding for the various cell populations of periodontium. Thus a more in-depth knowledge of cellular and matrix protein component of periodontium provides an excellent commencement for future advances [1].

This review compiles the basics of periodontal proteomics, currently used proteomic methods, proteomic biomarkers specific to periodontal structure, and applied proteomics in oral health and disease.

## 2. Need for a Periodontal Disease Indicator

The diagnosis of dynamic phase of disease, identifying patient at risk for periodontal disease, and focusing on early identification of microbial confront to host are tranquil for clinical investigations [3, 8, 9]. So there has been increasing interest in exploring protein biomarkers to get optimal, best possible, novel, and noninvasive approaches for the above stated causes [3, 8]. Thus the knowledge of periodontal disease indicator is a must to ultimately improve the clinical management of periodontal patients [8].

The roadblocks that have prevented the realization of periodontal diagnostics [10] are

- lack of definitive disease-associated protein and genetic biomarkers,
- expensive sampling method,
- lack of an accurate, easy-to-use diagnostic platform.

The novel expertise of miniaturization coupled with corresponding/analogous disease detection creates fundamental ways of detecting and diagnosing disease state by studies employing transcriptomics (oligonucleotide chips) known as the field of genomics. Genomics only can directly address the level of gene products present in cell state and has limited applications.

During the last few years, protein as a biomarker in periodontal disease has gained confirmation. The study of proteome, that is, composition, protein-protein interaction, systemic elucidation of protein, extracellular matrix interaction, and posttranslational modification, is in forefront of oral diagnosis. Proteomics thus provides systematic/comprehensive information about proteins in various tissues and organs [1] to have an excellent beginning for future advancements in the field of diagnostics.

## 3. Methods of Proteome Analysis

The tissue and cell complexity in the periodontium require the submission of more global experimental approaches for determining expression profiles [1]. Proteomic armamentarium contains a broad array of technical approaches [1]. For analysis of dissected periodontal tissues, sections through periodontium or cultured periodontal cells, fractionation of cells, and matrix followed by protein separation are the initial steps to proteomic study. The enumerated methods of proteome analysis are below.

- *ELISA (Enzyme Linked Immunosorbent Assay) *which is a tried and tested method for isolation and quantification of protein.
- In 1995, Randall Nelson pioneered the use of *immunoassays with mass spectrometry (MSIA).* To determine the set of proteins that have undergone posttranslational modification, antibodies can be developed which are specific to the modifications and can only recognize certain proteins.
- Recently, another approach has been developed called *PROTOMAP (Protein Topography and Migration Analysis Platform)* which combines Sodium Dodecyl Sulphate Poly Acrylamide Gel Electrophoresis (SDS-PAGE) with shotgun proteomics to enable detection of changes in gel migration such as those caused by proteolysis or posttranslational modification [11].
- More recent techniques such as *matrix-assisted laser desorption/ionization (MALDI)* [12] have been employed for rapid determination of proteins in particular mixtures.
- For analysis of complex protein mixtures derived from biological samples, *two-dimensional polyacrylamide gel electrophoresis *[13] remains an important technology.
- Nongel based proteome separation techniques to overcome the limitations of two-dimensional electrophoresis while preserving the ability to resolve complex protein and peptide mixtures before mass spectrometry analysis were developed.
- *Capillary electrophoresis* is an alternative to both two-dimensional electrophoresis for protein separation and to chromatography for peptide separation.

*Mass spectrometer-based proteomic analysis* is now being used more frequently in studies of interest to dental scientists including, for example, the analysis of Streptococcus mutans and the analysis of osteoblastic differentiation [14, 15]. The sequence of mass spectrometric analysis of an unknown mixture of proteins includes, first, the separation of proteins from the biological sample, digestion of the proteins, separation of the peptides, and then analysis of proteins by mass spectrometry and sequence analysis [1]. Mass spectrometers have improved ability to detect and characterize the amount of protein in biological samples. Still a major challenge is to determine how the complement of expressed cellular proteins—the proteome—is organized into functional, higher-order networks and to develop global protein—protein interaction networks on a cellular or tissue level [16].

**Figure 1:**
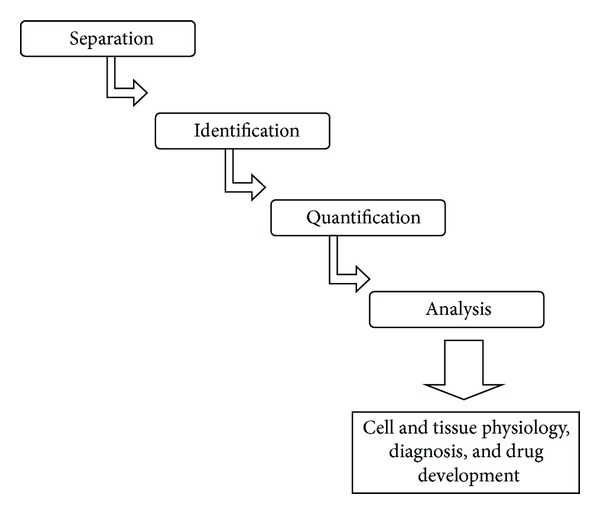
The major steps of separation to analysis of the fractionated proteins.

The following flow chart illustrates the major steps from the separation of the fractionated proteins till the determination of its sequence analysis [1] (Figure 1).

## 4. Types of Proteomics

### 4.1. Structural Proteomics

Study of proteomics is based on structural information of total repertoire for three-dimensional images for all proteins in an organism. This arises from analysis of unknown proteins such as protein-bound ligand or cofactor and is useful for functional description [1]. The identification of all proteins on a genome wide scale, determining their structural-functional relationships, and describing three-dimensional structures are the important hurdles in structural proteomics [17]. Functional and evolutionary protein relation which were not visible at sequence level are now possible with the advent of structural proteomics [18].

### 4.2. Interaction Proteomics

The functions of biological systems are dependent on interactions between their components. These interactions are ultimately determined by genetic elements and selection processes [19]. The sequencing of complete genomes provides information on the proteins responsible for cellular regulation. The different technique used for this includes yeast two-hybrid system, microassays, and affinity purification. This technology has been used for many different biological systems including, for example, identification of novel matrix metalloproteinase substrates that act to regulate inflammation [20].

### 4.3. Functional Proteomics

Types of proteins that indicate the function of proteins or how they are assembled into the molecular machines and functional networks that regulate cell behaviour [21] determine the functional proteomics. It is “focused to monitor and analyse the spatial and temporal properties of the molecular networks and fluxes involved in the living cells” [22]. It concentrates on the following two issues [23]:

- elucidation of biological functions of unknown proteins,
- cellular activity at molecular level.

## 5. Proteins of Periodontium

Periodontal proteomic markers range from salivary protein markers like Immunoglobulin G to bone remodeling protein markers [3]. These can be specific/nonspecific. Specific markers are immunoglobulins which characterize the presence of chronic or aggressive periodontitis. Among nonspecific markers are enzymes, proteins, mucins, histatin, lactoferrin, lysosomal peroxidase, and so forth. In addition, blood, GCF, serum, serum products, electrolytes, microorganisms, epithelial and immune cells, bacterial degradation products, lipopolysaccharides, and periodontal fibroblasts can be used for proteome analysis [24]. Biomarkers specific for periodontitis and any change in their composition could be diagnostic [3]. Comprehensive analysis and identification of proteomic contents in saliva, GCF, periodontal fibroblasts, and periodontal microbes are a necessary first step towards the discovery of periodontal protein markers for periodontal disease.

Possible potential periodontal biomarkers are as follows.

### 5.1. Immunoglobulins: (Ig A, Ig G, Ig M, and sIg A)

Immunoglobulins act as an innate defence mechanism of periodontium by interfering with adherence and metabolism of bacteria. The concentrations of salivary immunoglobulin (IgA, IgG, and IgM) are specific to periodontal pathogens which are higher in affected individuals. Following a successful periodontal treatment, the levels of these immunoglobulins in saliva are greatly reduced. Screening of saliva (noninvasive technique), especially for IgA, identifies individuals who have the potential to develop periodontal disease or those who are currently responding to a periodontopathogenic infection, thus forming a useful technique [7].

### 5.2. By-Products of Tissue Breakdown: (Collagen Telopeptides, Proteoglycans, Osteocalcin, Fibronection Fragments, and Bone Collagen Fragments)

Osteocalcin, osteonectin, and collagen telopeptidases and bone collagen are proteome biomarkers for bone homeostasis. These are connective tissue derived molecules. They are associated with local bone metabolism confined to periodontitis and systemic conditions like osteoporosis or metastatic bone cancers [8].

#### 5.2.1. Pyridinoline Cross-Linked Carboxyterminal Telopeptide of Type I Collagen

Pyridinoline, deoxypyridinoline, N-telopeptides, and C-telopeptides are class of degradation molecules which are released systemically during degradation of collagen matrix and bone resorption due to posttranslational modification of collagen. They have emerged as valuable proteome markers for bone turnover and are very specific for periodontal disease [25, 26]. These markers differentiate the active periodontal or peri-implant bone destruction from latent periodontal disease [27]. Palys et al. “related pyridinoline cross-linked carboxy terminal telopeptide of type I collagen (ICTP) levels to the subgingival microflora of various disease states on GCF and found ICTP levels differed significantly between health, gingivitis, and periodontitis subjects, and related modestly to several clinical disease parameters [28].” Depleted levels of ICTP subsequent to periodontal therapy imply that it is a good indicator of future alveolar bone and clinical attachment loss [29].

#### 5.2.2. Osteocalcin

It is the most abundant noncollagenous protein in bone which has specific calcium binding property [30]; it is synthesized mainly by osteoblasts and thus has a dominant role in bone remodeling [31–33].

Studies were done to evaluate its relationship with periodontal disease [34–36]. Kunimatsu et al. in the year 1993 reported “a positive correlation between GCF osteocalcin N-terminal peptide levels and clinical parameters in a cross-sectional study of periodontitis and gingivitis patients. Osteocalcin could not be detected in patients with gingivitis [34].” Later in the year of 1994, Nakashima et al. reported “significant GCF osteocalcin levels from periodontitis and gingivitis patients [36].” “On evaluation of a combination of the biochemical markers osteocalcin, collagenase, prostaglandin E2, *α*2-macro-globulin, elastase, and alkaline phosphatase, increased diagnostic sensitivity and specificity values of 80% and 91%, respectively, were reported” by Nakashima et al. in 1996 [35].

#### 5.2.3. Osteopontin (OPN)

It is noncollagenous calcium binding glycosylated phosphoprotein in bone matrix and is produced by several cells including osteoblasts, osteoclasts, and macrophages [37]. In 2001, Kido et al. demonstrated that “OPN level in GCF is significantly correlated with progression of periodontal disease [38].”

#### 5.2.4. Calprotectin

It is a key cytosol protein of leukocytes. Calprotectin has an important defence mechanism as it affects the activity of P. gingivalis [37]. Kido et al. in the year 1999 found “the concentration of calprotectin high in GCF of patient with periodontal disease” [39].

### 5.3. Host Factors

 
*Host response* includes monocytes, PMNs, macrophages, IL-1, TNF-*α*, and PGE_2_.
 
*Host cells* includes immune cells, interleukins, and periodontal ligament fibroblasts.
 
*Host derived enzymes* includes matrix metalloproteinases (MMPs), elastase, aspartate aminotransferase, cathepsin B, and acid phosphatase.

#### 5.3.1. Host Cells

**Figure 2:**
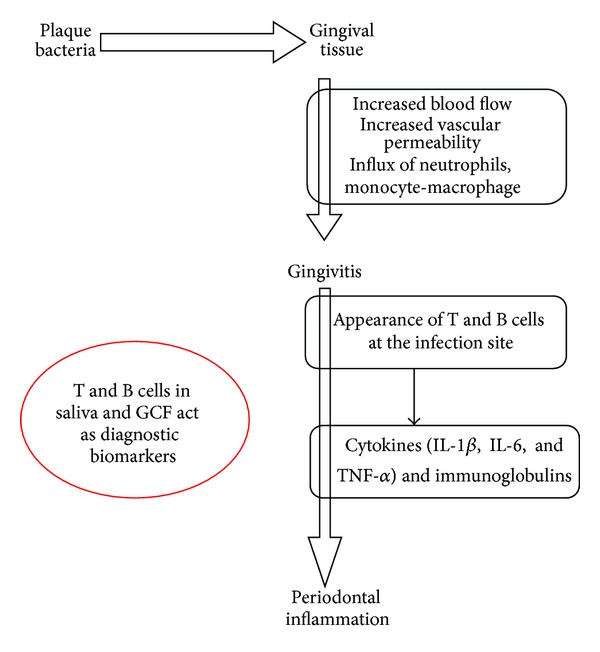
Role of host cells in periodontal inflammation.

Periodontal inflammation occurs in the gingival tissue in response to plaque bacteria biofilms (Figure 2) [40–43]. The cellular components of GCF include 70–80% granulocytes, 10–20% monocytes/macrophages, 5% mast cells, and 5% T lymphocytes [37]. Thus, the pathophysiological status of the periodontium in a site-specific manner can be assessed by proteome analysis of GCF samples [37].

#### 5.3.2. Inflammatory Cells

Periodontal inflammation in response to plaque bacteria biofilms subsequently induces an antigen-specific response [37]. Friedman and Klinkhammer developed the Orogranulocytes Migratory Rate (OMR) by standardized method of collecting and counting of leukocytes in saliva [44]. In a study by Khashu et al. in the year 1978, “the OMR was determined with sequential mouth rinse sampling in periodontitis patients and controls with results indicating that the OMR reflects the presence of oral inflammation and thus this measure can be used as a laboratory test [8].”

#### 5.3.3. Macrophages

Interleukins and prostaglandins are important inflammatory mediators released by macrophages and PMNs by the chemoattractant effects of lipopolysaccharide present in bacterial cell wall [45, 46] (Table 1) [46, 47].

#### 5.3.4. Periodontal Ligament (PDL) Fibroblast

Identification and characterization of PDL cellular components are important for understanding proteins. PDL is a dynamic tissue implying an intensive and balanced haemostasis regulated by cell-ECM interactions. As for protein synthesis in the functioning PDL, data have been obtained studying PDL fibroblast using immunological specific antibodies techniques [48]. Proteome analysis in functioning human PDL fibroblast has been studied and has revealed proteins that will broaden the basis for the future understanding of PDL cellular activities in health and disease [49].

**Figure 3:**
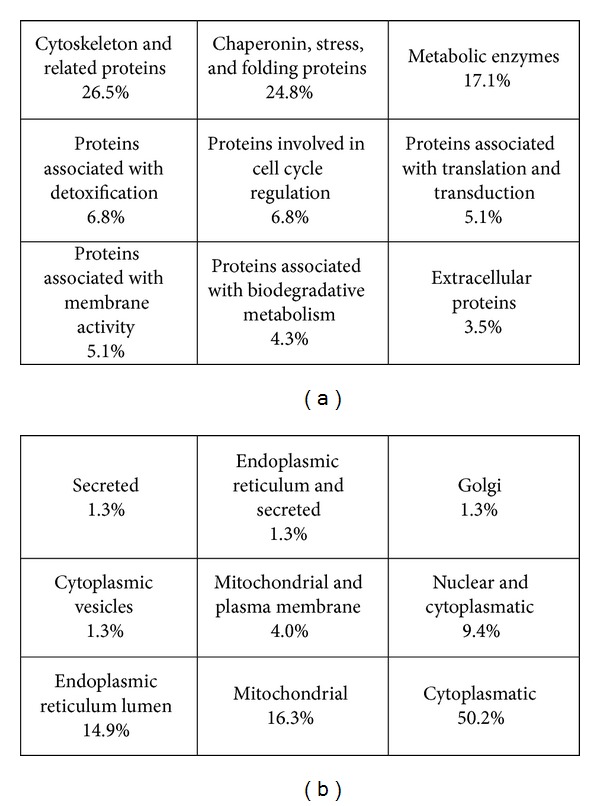
(a) Classification of proteins into functional groups according to biologic function; (b) subcellular location of proteins.

Unique biotopological functioning of PDL fibroblast and the application of proteomics on protein mixtures provide information about multiple gene products by means of expression levels and posttranslational modifications and are very powerful in the characterization of disease versus normal cells [50]. The proteomic analysis of the total proteins of PDL cells leads to the identification of 117 proteins that correspond to 74 different gene products creating a proteome map showing a variety of novel as well as expected proteins. The application of proteomic technology overcomes a number of limitations imposed by classic protein purification and characterization methods [49]. Detection of additional proteins may be achieved by applying a protein enriching technique including organelle fractionation, utilizing intensive solubilizing, and reducing agents and by using chromatographic separation methods. The metabolic needs and capabilities of the PDL fibroblast are demonstrated by the identification of 20 spots as metabolic enzymes. Analyzing the subcellular distribution of the identified proteins, it was found that 50.2% are cytoplasmic and another important set of identified protein groups was composed of endoplasmic reticulum 14.9% and mitochondrial 16.3%. Membrane associated proteins constituted 4%, and the cytoplasmic vesicles constituted 1.3% (Figure 3).

Figure 3(a) shows the identified proteins classified into functional groups according to their biological functions and Figure 3(b) shows subcellular location of the proteins as annotated in the SWISS-PROT database. No annotation existed for 35% of the proteins.

#### 5.3.5. Neutrophils

Neutrophils are the first line of host defence against periodontopathogenic bacteria. Hydrolytic neutral enzymes (elastase, cathepsin G, myeloperoxidase, lysozyme, hydrolases, lactoferrin, and neutrophil collagenase like MMP-8 and MMP-9) are present in neutrophlic granules [37]. *β* glucuronidase is a lysosomal enzyme that acts as a marker for release of primary granule from PMNs [37]. An increased level of enzyme esterase is seen in periodontally compromised individuals and also during calculus formation [37].

#### 5.3.6. Matrix Metalloproteinases (MMPs)

Host cell-derived enzymes such as matrix metalloproteinases (MMPs) are an important group of neutral proteinases implicated in the destructive process of periodontal disease that can be measured in GCF [51]. The neutrophils are the major cells responsible for release of MMP and more importantly MMP-8 (collagenase-2) and MMP-9 (gelatinase-B) which is a concern to a periodontist as it is released during acute stages of periodontal disease [52]. Inflamed human gingiva and GCF in subjects with adult periodontitis have increased levels of MMPs [46, 53]. MMP-8, being a key enzyme in extracellular collagen matrix degradation, increased levels are seen in peri-implant sulcular fluid from peri-implantitis lesions and hence can be employed as a biomarker in the active phase of peri-implant disease [54]. Thus, apart from being a disease severity indicator, MMP-8 also measures disease activity [3].

Gelatinase (MMP-9) degrades collagen intercellular ground substance and may serve as a guide in periodontal treatment monitoring as its level is higher in GCF of the patients with chronic periodontitis than in healthy patients [8]. Collagenase-3 or MMP-13 is another collagenolytic MMP with exceptionally wide substrate specificity and also a role in peri-implantitis. MMP-13 may be useful for diagnosing and monitoring the course of periodontal disease and for tracking the efficacy of therapy [55]. It was shown that elevated levels of both MMP-13 and MMP-8 correlated with irreversible perioimplant vertical bone loss around loosening dental implants [56]. MMP-2 is secreted by gingival fibroblasts and the crevicular MMP-2 levels were seen to be lower in gingivitis and periodontitis conditions [57]. According to a study by Rai et al. in 2008, they showed that “the levels of MMP-8, MMP-2 and MMP-9 were highly correlated to probing depth, and bleeding on probing and concluded that MMP-8, MMP-2 and MMP-9 are biomarkers of periodontal disease and aid in early detection of periodontitis or gingivitis [58].”

#### 5.3.7. Nitric Oxide

**Figure 4:**
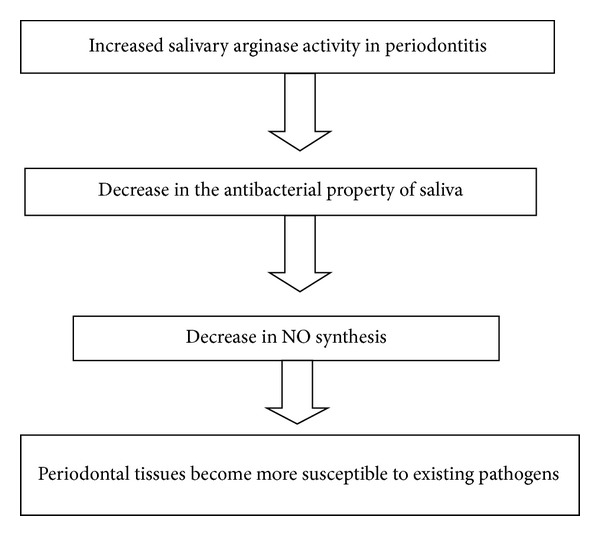
Role of nitric oxide (NO) in periodontal inflammation.

Nitric oxide [NO] is a free radical with important cellular functions and is produced and released from human neutrophils and macrophages [59]. NO is synthesized from the conversion of L-arginine to L-citrulline by nitric oxide synthase [NOS]. Arginase, an arginine-depleting enzyme, can compete with NOS for the common substrate L-arginine and thus inhibit NO production (Figure 4) [59, 60].

#### 5.3.8. Cathepsin B

Cathepsin B, a cysteine protease whose source in GCF is mainly from macrophages, contributes to periodontal tissue destruction by proteolytic activation of neutrophil procollagenase (Promatrix metalloproteinase-8) [37]. The levels of cathepsin B were observed to increase in periodontitis when compared to gingivitis, despite similar GCF flow, and thus differentiate chronic gingivitis from periodontitis [61].

#### 5.3.9. Aspartate Aminotransferase Enzyme (AST)

AST is a tissue destruction biomarker released from necrotic cells in GCF and is associated with periodontitis severity [62, 63]. Persson et al. reported “significant associations between GCF levels of AST and clinical measurements, and a test system, the Periogard periodontal tissue monitors (PTM) been developed [64, 65].”

#### 5.3.10. Alkaline Phosphatase (ALP) & Acid Phosphatase (ACP)

They are membrane-bound glycoproteins engaged in preservation of alveolar bone and renewal of the periodontal ligament [37]. Nakashima et al. demonstrated that “high levels of ALP preceded clinical attachment loss and that the total amount of ALP in GCF was significantly higher in active sites [35].” The mixed whole saliva of adults with periodontal disease was shown to reveal the highest enzyme activities with ALP than that of healthy individuals [66]. Hence it was concluded that salivary ALP can be considered as a useful marker for monitoring periodontal disease [37]. A study on salivary enzymes and calculus formation found a significant association between salivary ACP and calculus formation [67].

### 5.4. Microbial Factors

**Figure 5:**
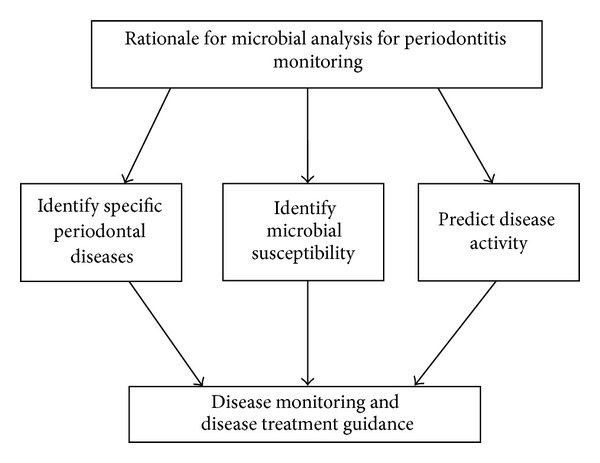
Rationale for microbial analysis for periodontitis monitoring.

Various bacterial species localized in subgingival plaque, from which only few play a causal role in the pathogenesis of periodontal diseases in the susceptible host [68]. Specific bacterial species of interest in periodontal pathogenesis are *T. forsythensis*, *P. gingivalis*, *T. denticola*, and *A. actinomycetemcomitans* [69]. Members of the “red complex” of periodontal pathogens (*T. forsythensis*, *P. gingivalis*, and *T. denticola*) exhibit BANA activity (benzoyl-DL-arginine-naphthylamide) and are strongly correlated with periodontal activity [70–72]. The basic rationale for microbial analysis for periodontitis monitoring is to target pathogens implicated in disease [73] (Figure 5). Taba Jr. et al. in the year 1998 tested the presence of periodontal pathogens in whole saliva in relation to occurrence of the microorganisms in subgingival plaque. They found that “using polymerase chain reaction, a fair agreement was found between the presence of *P. gingivalis*, *P. intermedia* and *T. denticola* in whole saliva and in periodontal pocket samples [74].”

#### 5.4.1. *A. actinomycetemcomitans*

It is known that an insight into the subgingival microbial flora of the dental plaque biofilm has led to improved understanding of periodontitis and also mechanisms responsible for its systemic link [75]. Virulence factors of *A. actinomycetemcomitans* can be delivered into human cells via outer membrane vesicles (OMVs) or by free-soluble surface components with proinflammatory activity [76, 77]. Abundance production of both OMVs and free-soluble surface material is seen in the plaque; they form a significant source of inflammatory stimulants along with the planktonic bacteria in the haemopoietic system [78].

Alugupalli et al. reported that “Lactoferrin interacts with *A. actinomycetemcomitans*, which is a causative microorganism in aggressive periodontitis and its colonization may occur more readily in an environment containing lactoferrin with low iron levels and depressed level of iron found in lactoferrin may be resulted from both the iron-sequestering pathogenic bacteria and reduced capacity of lactoferrin to bind iron in the saliva of aggressive periodontitis patients [37, 79].”

*P. gingivalis* is implicated in the immune and inflammatory host response in periodontal disease as it shows the greatest proteolytic activity through peptidases, elastases, trypsin-like proteases, and collagenases that can be monitored by proteome analysis of GCF [74].

### 5.5. Phenotypic Markers

#### 5.5.1. Epithelial Keratin

For epithelial cell function in periodontal disease and periodontal diagnosis, specific keratin antigens in saliva and detection of keratins by monoclonal antibodies may have diagnostic value in detection of epithelial dysplasia, oral cancer, odontogenic cysts, and tumours [80]. The phenotypic markers for junctional and oral sulcular epithelia can be used as indicators of periodontal disease [8]. McLaughlin demonstrated that “the keratin concentration in GCF was significantly higher at sites exhibiting signs of gingivitis and periodontitis compared with healthy sites [81].”

#### 5.5.2. Fibronectin

Fibronectin is a glycoprotein which mediates adhesion between cells [8]. Salivary fibronectin is reduced in periodontitis as *P. gingivalis* fimbriae bind to fibronectin [37].

### 5.6. Volatile Compounds

Volatile compounds are hydrogen sulphide, methyl mercaptan, picolines, and pyridines.

According to Rosenberg and McCulloch in 1992, “volatile sulphur compounds, primarily hydrogen sulfide and methylmercaptan, are associated with oral malodour [82].” Salivary volatiles can be used as possible diagnostic markers in individuals with moderate to severe periodontitis, although no specific association between levels of volatiles and periodontal status has been reported [8].

### 5.7. Hormones

#### 5.7.1. Cortisol

In individuals exhibiting severe periodontitis, a high level of stress with emotion-focused coping, higher salivary cortisol levels were observed exerting a strong inhibitory effect on the inflammatory process and immune response [8, 83].

### 5.8. Ions

#### 5.8.1. Calcium

Calcium (Ca) is the ion that has been most intensely studied as a potential marker for periodontal disease in saliva. Sewón et al. showed in their studies that “higher concentration of salivary Ca and the saliva Ca to phosphate ratio were higher in individuals affected by periodontal disease and thus concluded that an elevated Ca concentration in saliva was characteristic of patients with periodontitis [84, 85].”

### 5.9. Lactoferrin

Groenink et al. demonstrate that “it is strongly up-regulated in mucosal secretions during gingival inflammation and is detected at a high concentration in saliva of patients with periodontal disease compared with healthy patient [86].”

### 5.10. Platelet Activating Factors

Platelet activating factor [PAF] is a potent phospholipid inflammatory mediator. Rasch et al. have demonstrated “significant correlation between salivary platelet activating factor (PAF) levels and the extent of periodontal disease [87].”

## 6. Future Trends

Several possibilities for further application of proteome map in biotechnology and health care applications, especially in the field of diagnostics, exist. Huge amount of research activity has already been done to expose the role of oral and salivary fluids in oral diagnostics. Recent advances in HIV diagnosis, for example, OraSure, OraSure Technologies, Bethlehem, Pennsylvania, which collects HIV-1 antibodies from gingival tissues using oral mucosal transudate, are entirely based on proteome analysis.

Several researchers have focused on genetic single nucleotide polymorphisms in the study of periodontal disease. A genetic susceptibility test is available for severe chronic periodontitis (Interleukin Genetics, Waltham, Massachusetts). It works by detection of two types of IL-1 genetic alleles, IL-1*α* + 4845 and IL-1*β* + 3954 [88]. Individuals identified as “genotype positive,” or are found to have both of these alleles, are more likely to have the phenotype of overexpression of this gene. High levels of GCF and salivary IL-1 predispose the patient to the severe form of chronic periodontitis by way of a hyperinflammatory response to bacterial challenge. In this way, proteomics has been found to be applicable in the prediction of predisposition to periodontitis in certain patient populations [89]. Socransky et al. researched IL-1 gene polymorphisms in periodontitis patients by linking previous findings regarding the association of IL-1 polymorphisms and severity of adult periodontitis with microbial species found in IL-1 genotype-negative versus IL-1 genotype-positive patients and hence concluded that “those who were IL-1 genotype positive tended to have higher levels of the more damaging microbial species (red and orange complex organisms) associated with periodontal inflammation [90].”

Salivary immune components have also been studied extensively for oral health, also immunoglobulin subclass, immunoglobulin isotypes, and antibody levels [91–93]. Other salivary constituents that have been investigated for diagnostic uses include epithelial keratins^98^, occult blood^99^, salivary ions such as calcium and phosphates, and serum markers such as cortisol [94–98].

However, as protein expression and posttranslational modifications are dynamic processes, particularly in the periodontium, identification and quantification of proteins alone are not sufficient to comprehend functional changes. New technologies will be needed to enable combinations of metabolic labelling and identification as well as quantification and measurement of synthesis rates of proteins [99].

## 7. Summary

In the area of oral disease diagnosis, a steady growing trend in the last 2 decades to develop tools to detect and monitor periodontitis has been seen. From traditional physical measurements such as periodontal probing to new, sophisticated genetic susceptibility analysis and molecular assays for the detection of biomarkers on the whole spectrum of the disease process, substantial improvements have been made on the understanding of the mediators implicated on the initiation and progression of periodontitis. At the same time, evolution in this field has promoted the discovery of new biomarkers and the development of new therapeutic approaches mainly using host modulation. Further, new diagnostic technologies such as nucleic acid and protein microarrays are under development for risk assessment and comprehensive screening of biomarkers. Proteomics can provide comprehensive and systematic information about proteins in a wide array of tissues and organs. The recent advances are leading to the development of more powerful diagnostic tools for practitioners to optimize their treatment predictability.

## Figures and Tables

**Table 1:** Interleukins and prostaglandins.

IL-1*β*	PGE_2_
(i) Proinflammatory cytokine plays a key role in the etiology of periodontal disease	(i) Involved in the pathogenesis of periodontal diseases
(ii) Stimulates induction of molecules to amplify tissue response	(ii) “A difference of concentration of PGE_2 _in GCF was shown in patients with gingivitis and periodontitis” by Offenbacher et al. in the year 1986 [47].
(iii) The level of cytokines proportionately correlates with periodontal parameters adjusting for the confounders	
(iv) Increasing IL levels increase the risk of periodontal disease by 45-fold [46]	
